# Linking nutrient availability and community size to stochasticity in microbial community assembly

**DOI:** 10.1093/femsec/fiaf110

**Published:** 2025-10-28

**Authors:** Berenike Bick, Theresa Lumpi, Eva S Lindström, Silke Langenheder

**Affiliations:** Department of Ecology and Genetics/Limnology, Uppsala University, Uppsala 75236, Sweden; Department of Aquatic Sciences and Assessment, Division of Microbial Ecology, Swedish University of Agricultural Sciences, Uppsala 75007, Sweden; Department of Ecology and Genetics/Limnology, Uppsala University, Uppsala 75236, Sweden; Department of Ecology and Genetics/Limnology, Uppsala University, Uppsala 75236, Sweden

**Keywords:** bacterioplankton, community ecology, ecological stochasticity, microbial communities, microcosm experiment

## Abstract

Both deterministic (e.g. species–environment interactions) and stochastic processes (e.g. random birth and death events) shape communities, but it remains poorly understood, which environmental conditions promote stochasticity. Here, we investigated interactive effects of nutrient availability and community size on stochasticity in order to predict how eutrophication and biomass loss shift the balance between predictable and random community dynamics. For this, we used freshwater bacterial communities in a microcosm experiment, where communities were diluted to varying sizes and exposed to low, intermediate, and high nutrient concentrations. Stochasticity was estimated with null modelling and as beta-diversity among replicate communities. At low nutrient concentrations, deterministic processes dominated, especially in smaller communities, which had the lowest diversity and abundance. Whereas, higher nutrient concentrations increased stochasticity. In contrast to theoretical predictions, this was particularly the case in larger communities with the highest diversity and abundance, likely due to stochastic initial growth. The findings underline how nutrient availability and community size jointly influence stochastic assembly processes, with important consequences for bacterial diversity and ecosystem functioning under environmental change.

## Teaser

This study shows that nutrient availability and community size jointly determine whether freshwater bacterial communities are shaped more by deterministic or stochastic processes, with low nutrients favouring deterministic assembly and high nutrients promoting stochasticity, especially in larger, more diverse communities.

## Introduction

The four main assembly processes that determine spatial and temporal differences in community composition, i.e. beta-diversity, are selection, drift, dispersal, and diversification (Vellend 2016). These processes can have deterministic as well as stochastic components. Selection is a purely deterministic process, in which organisms in a community are selected by fitness differences due to, e.g., abiotic factors (Nemergut et al. 2013). Dispersal and diversification, on the other hand, can be comprised of both stochastic and deterministic components (Zhou and Ning 2017). On the contrary, drift, i.e., the probability of a species in a community to go extinct before reproducing due to randomly occurring birth and death events, is purely stochastic (Vellend 2016). Moreover, priority effects, i.e., the arrival sequence of species shaping the assembly of a community, is also viewed as a stochastic process (Fukami 2015).

Community assembly processes in microorganisms have been the subject of debate for decades, and so far, studies have mainly focused on deterministic processes (Zhou and Ning 2017). Thus, stochastic processes, like drift, have received less attention, even though it has been shown that they can contribute largely to assembly in, for example, aquatic microbial communities (Liu et al. 2019, Zhao et al. 2023). Studies have shown that the importance of stochasticity in community assembly can increase over time, leading to enhanced dissimilarity among initially identical communities (Zhou et al. 2013, Vellend et al. 2014). Moreover, it is known that communities with low alpha-diversity are more likely to be stochastically assembled (Chase and Myers 2011, Vieira et al. 2022), and that stochasticity is enhanced under dispersal limitation when environmental conditions are similar among habitats (Evans et al. 2017, Albright et al. 2019). Although quantifying stochasticity presents significant challenges, a variety of methodological approaches have been developed to do so. For instance, stochasticity can be measured as beta-diversity among replicate communities (Vellend et al. 2014), or by using null model approaches that determine the relative importance of deterministic versus stochastic processes (Chase and Myers 2011, Zhou and Ning 2017, Ning et al. 2019).

Studying stochasticity becomes particularly important when ecological systems experience disturbances. Environmental disturbances can reduce habitat size or community biomass, leading to declines in species richness and abundance, and ultimately increasing the community’s vulnerability to stochastic processes (Chase 2010), especially in enclosed systems experiencing dispersal limitation (Zhou et al. 2014). As community size decreases with such disturbances, each individual cell has proportionally a larger impact on the community compared to larger, undisturbed communities and therefore, random birth and extinction events can shift community composition and overrule selection pressure (Orrock and Fletcher 2005, Orrock and Watling 2010, Hubbell 2011, Nemergut et al. 2013, Siqueira et al. 2020). As many microbial taxa in a community can be low in abundance, they are especially prone to stochastic changes in community assembly (Nemergut et al. 2013, Liu et al. 2021). Moreover, it has been shown that enhanced nutrient availability can promote stochastic community assembly (Zhou et al. 2014, Ren et al. 2017, Cao et al. 2021). This effect is thought to arise because increased availability of resources reduces competition, which weakens selection and enhances growth among species, which can promote random population dynamics and priority effects (Chase and Leibold 2002, Chase 2010, Zhou et al. 2014).

Only a few studies have so far investigated interactive effects of different, simultaneously occurring factors on community assembly processes (Houseman et al. 2008). For example, it is not fully understood how a reduction of community size, e.g., due to a disturbance, and an increase in nutrient availability combined affect community assembly in bacteria (Zhou and Ning 2017), in particular in aquatic ecosystems. Therefore, this study aims to experimentally investigate combined effects of variations in community size and different nutrient concentrations on stochasticity in natural freshwater bacterial communities. Here, ecological stochasticity is measured as beta-diversity among replicate communities and tested with null modelling. We hypothesized that, (i) communities become more dissimilar over time, (ii) small communities show lower richness and are to a greater extent stochastically assembled, (iii) stochasticity is more important at high nutrient concentrations, because higher bacterial production and growth stimulate random population dynamics, and (iv) there are interactive effects between community size and nutrient concentrations, and stochasticity is strongest when small communities encounter high nutrient concentrations, and weakest in large communities at low nutrient concentrations.

## Methods

### Experimental design

**Figure 1.**
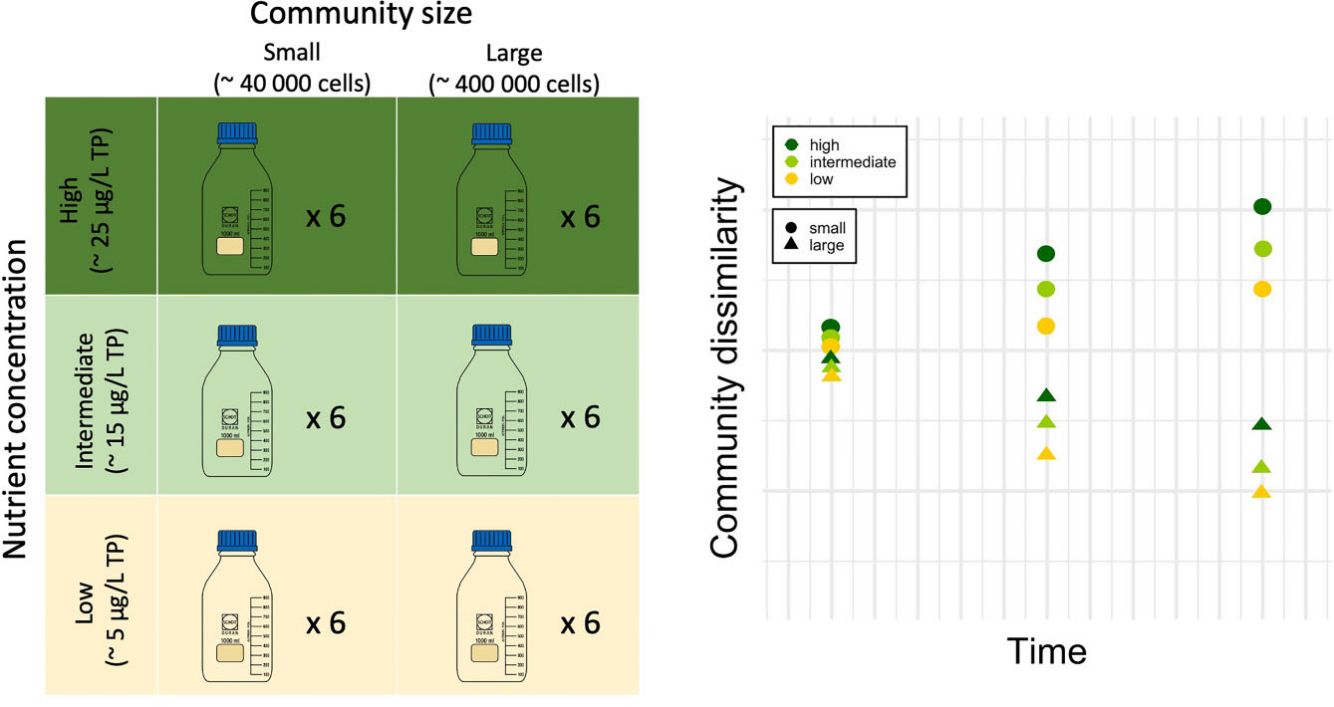
(a) Experimental set-up based on a 2 × 3 factorial design with two community sizes (small, and large) and three nutrient concentrations (low, intermediate, and high). Each treatment had six replicate microcosms. Small community size microcosms contained 10 mL and large community sizes 100 mL of bacterial inoculum and 490 mL and 400 mL of sterile lake water medium, respectively. Low nutrient concentrations encompassed 5 µg/L, intermediate 15 µg/L, and high 25 µg/L of total phosphorus (TP). (b) Conceptual figure of the expected results for community dissimilarity in the different treatments, for details see text. .

In order to test the hypotheses of this study, a microcosm laboratory experiment with a natural freshwater bacterial community was conducted over 13 days. Borosilicate glass bottles (1 L) were filled with 500 mL of a mixture of sterile medium and bacterial inoculum. Six treatments were set up, including two community sizes (small, and large), i.e., the ratio of bacterial inoculum to experimental medium, and three different nutrient levels (low, intermediate, and high), each replicated six times (Fig. 1). All microcosms, were kept at 20°C for the duration of the experiment, including nine contamination controls, which contained only sterile medium (*n* = 45). Samples for bacterial abundance were taken every second day, and samples for biomass production and community composition were taken at five time points throughout the experiment (days 1, 4, 7, 10, and 13) from each microcosm. Differences in community size were established by varying the number of individuals in a community (i.e., the number of cells inoculated at the start of the experiment). Stochasticity was measured as the variation in community composition between initially equivalent communities developing under identical environmental conditions and dispersal limitation. This was analysed as beta-diversity among replicate communities and tested with null modelling. Bacterial production was estimated in two ways: firstly, as biomass production using leucine incorporation, and, secondly, by changes in bacterial abundance over time.

### Preparation of the experimental medium

For the experimental medium and bacterial inoculum, a total of 80 L of water were taken from oligotrophic (10 µg/L total phosphorus = TP, 800 µg/L total nitrogen = TN, and 17.6 mg/L total organic carbon = TOC) lake Siggeforasjön, Sweden (59° 58′ 42.1″ N 17° 09′ 26.7″ E) on 8 October, 2021. For sterilization, water was filtered, with a tangential flow filtration unit with a pore size of 0.1 µm (Cytiva Europe GmbH), and autoclaved at 121°C for 20 min. The pH of the autoclaved medium was adjusted with HCl (0.1 M) to match original lake conditions (pH 6.6). In order to acquire a nutrient gradient, the medium was split into three batches: Low nutrient concentrations (~5 µg/L TP, 480 µg/L TN, and 20 mg/L, TOC), intermediate concentrations (~15 µg/L TP, 480 µg/L TN, and 20 mg/L TOC) and high concentrations (~25 µg/L TP, 700 µg/L TN, and 20 mg/L TOC). Compared to the original lake conditions, bacterial communities experienced a decrease in TP in the low nutrient treatment and an increase in TP in intermediate and high nutrient treatments. These nutrient concentrations were measured in the experimental medium, and concentrations in the actual microcosms were calculated, which is why approximate concentrations are given here. The nutrient concentrations chosen in this study align with natural phosphorus concentrations in Swedish and European lakes (Huser and Fölster 2013, European Environment Agency 2024). Na_2_HPO_4_ was added to the intermediate and high nutrient media to increase TP levels, whereas no nutrients were added to the low nutrient treatments. Moreover, NH_4_Cl was added to the high nutrient medium to prevent nitrogen depletion in the microcosms according to the Redfield ratio 106C:16N:1P (Redfield 1958). Carbon adjustments were not necessary to match Redfield ratio.

### Preparation of the bacterial inoculum

For the preparation of the bacterial inoculum, lake water was filtered through Whatmann GF/F filters (0.7 µm) to remove grazers. Prior to the start of the experiment, the bacterial inoculum was pregrown at 20°C in the dark for 10 days until reaching stationary growth (Fig. S1). At the start of the experiment on 19 October 2021 (day 0), microcosms were set up with 2% (10 mL) or 20% (100 mL) of bacterial inoculum for the small (with an initial community size of around 40 million cells) and large community sizes (with an initial community size of around 400 million cells), respectively. The remaining volume (490 mL for small and 400 mL for large community sizes) was filled with either low, intermediate, or high nutrient medium, with nutrient concentrations modified to account for the different community size volumes. Additionally, nine control microcosms, containing 500 mL of medium (three replicates for each nutrient concentration), were included to account for potential contaminations throughout the experimental procedures, and were handled in an identical manner to the remaining experimental units. There was no bacterial growth detected in the contamination controls.

### Bacterial abundance and biomass production

All microcosms were homogenized by mixing prior to sampling and handled under sterile conditions in a laminar flow bench. Every second day, 950 µL of each microcosm were sampled for bacterial abundance, fixed with formaldehyde solution (1.85% formaldehyde in the sample) and stored at 4°C overnight. The following day, samples were stained with 1.25 µM fluorescent nucleic acid stain SYTO13 (Invitrogen) and cell abundance was measured using the CytoFLEX flow cytometer (Beckman Coulter) (Giorgio et al. 1996). If necessary, bacterial abundance samples were diluted with phosphate-buffered saline (0.01 M). Bacterial biomass production (see Supplementary Methods S2 for detailed description) was measured by leucine (^3^H) incorporation into bacterial protein (Kirchman et al. 1985, Smith and Azam 1992). Briefly, samples were taken at five time points throughout the experiment (days 1, 4, 7, 10, and 13) and incubated with leucine (L-[3, 4, 5 -3H], Perkin Elmer). After incubations, samples were measured in disintegrations per minute with a liquid scintillation counter (Hidex 600 SL, counting time: 300 sec, coincidence time: 35 ns) and converted into biomass production rates (Kemp et al. 2018).

### Bacterial community composition

To determine bacterial community composition, a volume of 100 mL was sampled per microcosm at five sampling points (days 1, 4, 7, 10, and 13), filtered onto a 0.2 µm Supor membrane filter (Pall) and stored at −80°C until further processing. To compensate for loss of volume and nutrient exchange, each microcosm received 100 mL of the respective sterile medium at these five samplings. Replicate 1 of the intermediate nutrient treatment and the small community size on day 13 was lost during sampling. For further analysis of bacterial community composition, DNA was extracted from the Supor membrane filters with the DNeasy PowerSoil Pro Kit (Qiagen) and polymerase chain reactions (PCRs) for amplicon library preparation were run in a two-step protocol based on Vass et al. (2021). Detailed description of the library preparation can be found in the Supplementary Methods (Supplementary Methods S1). In brief, the first PCR was run in triplicates with custom bacteria forward primer 341F (5′-CCTACGGGNGGCWGCAG-3′) (Herlemann et al. 2011) and reverse primer 805R (5′-GACTACNVGGGTATCTAATCC-3′) (Apprill et al. 2015). The second PCR was run without replicates, and individual Illumina adapter combinations were attached to the amplicons. Pooled samples, were sequenced at SciLifeLab SNP&SEQ Technology Platform (Uppsala University), using the Illumina MiSeq v3 sequencing protocol. The 16S rRNA sequences were demultiplexed and sequence-pair assembled at SciLifeLab, Uppsala. Primer removal, quality filtering (maximum error = 2), and trimming (quality score = 20), were carried out with cutadapt, version 4.8. Sample inference, merging of denoised reads and removal of chimeric sequences was done with dada2 (version 1.30.0) in R (version 4.3.1). Amplicon sequencing variants (ASVs) were taxonomically assigned against the SILVA database (version 138.1; Quast et al. 2013). This resulted in 608 ASVs in 178 samples in total, with an average sequencing depth of 37 998 reads. Analysis was done on resources provided by the Uppsala Multidisciplinary Center for Advanced Computational Science (UPPMAX).

### Statistical analyses

One sample (replicate 2 of the high nutrient and large community size treatment on day 4) had to be excluded from data analysis, because it had insufficient sequencing depth (< 10 000 reads). All statistical analyses were carried out in R (version 4.4.2). For ASV richness and Pielou evenness, ASV abundances were normalized by rarefaction to the minimal sequencing depth of 11 707 reads per sample (Fig. S2), using *rrarefy* in package vegan (version 2.6–8; Oksanen et al. 2017, Gloor et al. 2017). Pielou evenness was estimated as Shannon diversity divided by the natural logarithm of ASV richness using the package vegan (Pielou 1966). We evaluated how nutrient concentration and community size influenced bacterial abundance, biomass production, ASV richness, and Pielou evenness using linear mixed-effects models. The models included a random intercept for Replicate ID and accounted for within-replicate temporal autocorrelation across experimental days using a first-order autoregressive structure (corAR1) with day in the experiment as the time index. Experimental day was used only to specify the correlation structure and was not included as a fixed effect. Models were fit with *lme* from the package nlme (version 3.1–166), tests of fixed effects used type II Wald χ² tests using Anova in the car package (version 3.1–3). We also tested the effects of nutrient concentration, community size, and time point (treated as a categorical factor) on pairwise Aitchison distances and Bray–Curtis-based NST using linear mixed-effects models. The model included Replicate ID as a random intercept and accounted for temporal autocorrelation within replicates using a first-order autoregressive correlation structure (corAR1). The model was fit using *lme* and fixed effects assessed using type II Wald chi-square tests implemented in the *Anova* function. Normality of residuals was checked in all models by plotting Q–Q plots. As amplicon data is compositional and constrained by sequencing depth, ratios between taxa are more reliable than absolute abundances (Gloor et al. 2017). Therefore, Aitchison distances, compared to other dissimilarity metrics, due to working in log-ratio space, provides more robust ecological interpretations of proportional change in community composition (Aitchison et al. 2000). In order to test community composition differences between replicates within each treatment, pairwise Aitchison distances were calculated as the Euclidean distance of clr transformed data using *transform* in the package microbiome and *vegdist* from package vegan. To visualize treatment effects on community data in an ordination, we performed principal coordinate analyses (PCoA) on overall Aitchison distances for each experimental day 1, 4, 7, 10, and 13 separately, using *pcoa* in package ape (version 5.8; Paradis and Schliep 2019). Permutational multivariate analysis of variance (PERMANOVA) was conducted separately for each time point with *adonis2* in package vegan with 999 permutations. To test the relative importance of stochastic and deterministic processes in community assembly, normalized stochasticity ratios (NST) were computed with *tNST* using Bray–Curtis similarity and a proportional-fixed null model with the package NST (version 3.1.10; Ning et al. 2019). Randomizations were performed within each treatment and time point, so that null expectations were generated independently for each treatment and time point, and each analysis included 999 randomizations (rand = 999). NST quantifies the relative importance of deterministic compared to stochastic processes, where values of 0 and 1 represent purely deterministic and purely stochastic assembled communities, respectively. Hence, NST values above 0.5 indicate that stochastic processes, and values below 0.5 indicate that deterministic processes predominantly determine variation in community composition. Bootstrap analysis (999 runs) was performed using *nst.boot* in the package NST to retrieve values for plotting boxplots.

## Results

### Bacterial abundance and biomass production

**Figure 2.**
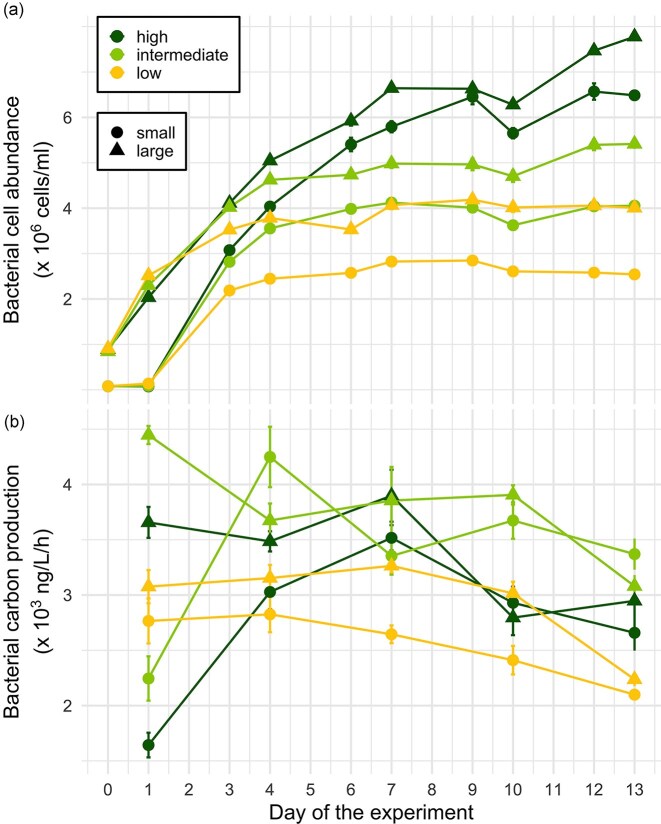
(a) Bacterial abundance and (b) bacterial biomass production during the experiment in treatments with different nutrient concentrations (low, intermediate, and high) and community sizes (small, and large). Points represent mean values and error bars standard errors.

Nutrient concentration (*P* = .051) and community size (*P* = .094) showed statistical trends in their effects on bacterial abundance, whereas their interaction had no detectable effect (Table S2). At the start of the experiment, bacterial abundance increased across all treatments (Fig. 2a), but small communities showed notably lower overall abundance and a delayed growth onset compared to larger ones. While growth plateaued under low and intermediate nutrient concentrations, bacterial communities in high nutrient concentration continued to grow and increased in abundance throughout the experiment. Consequently, the highest bacterial abundance was observed in large communities with high nutrients, whereas small communities under low nutrient concentrations maintained the lowest cell numbers. Similarly, bacterial biomass production was significantly shaped by nutrient concentration and community size, but not by their interaction (Table S2). Initially, large communities exhibited greater biomass production than small ones, and most treatments peaked in biomass production in middle of the experiment before declining (Fig. 2b). Communities exposed to intermediate nutrient concentrations show the highest biomass production rates, while low nutrient concentrations consistently had the lowest production rates.

### Alpha diversity

**Figure 3.**
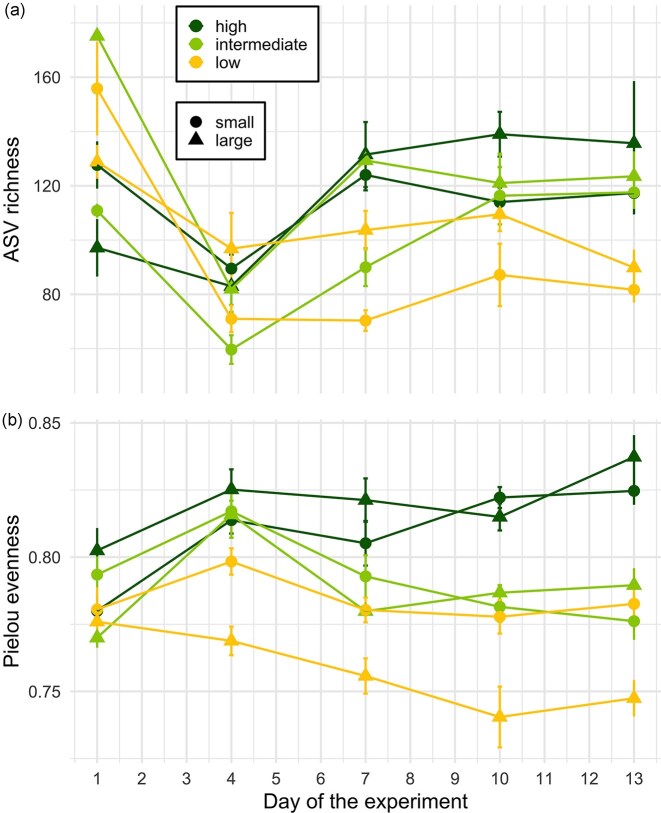
(a) ASV richness and (b) Pielou evenness on days 1, 4, 7, 10, and 13 of the experiment in treatments with different nutrient concentrations (low, intermediate, and high) and community sizes (small and large). Data is based on rarefied 16S rRNA gene amplicon sequences. Points represent mean values and error bars show standard errors.

The highest number of unique ASVs (*n* = 79) was found in large communities under high nutrient concentrations, whereas the lowest (*n* = 38) occurred in small communities at intermediate nutrient concentrations (Fig. S5). Shared ASVs followed a different pattern, with the greatest overlap (*n* = 36) found in large communities at intermediate and the least (*n* = 25) in large communities at high nutrient concentrations. Alpha-diversity, both richness and evenness, was significantly influenced by nutrient concentration and community size, but only evenness was also significantly influenced by their interaction (Table S2). Richness declined in all treatments early in the experiment, reaching a minimum around day 4, before gradually increasing again toward the end (Fig. 3). The lowest richness values occurred in low nutrient treatments, particularly in small communities. In contrast, the highest richness was observed in large communities exposed to high nutrient concentrations. Contrary, evenness increased across all treatments at the start of the experiment, peaking around day 4 in low and intermediate nutrient concentrations. After this peak, evenness declined in these treatments, while continuing to rise in high nutrient concentrations until the end of the experiment.

### Changes in bacterial community composition

**Figure 4.**
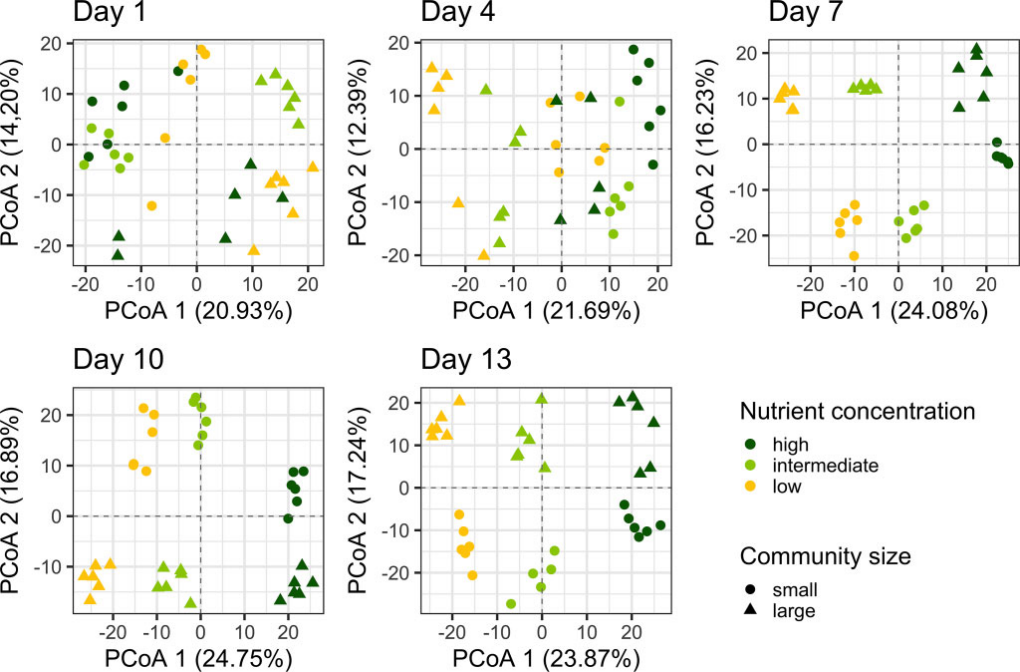
PCoA showing differences in bacterial community composition (based Aitchison distances using 16S rRNA gene amplicon sequencing data) depending on community size (small, large) and nutrient concentrations (low, intermediate, and high) on days 1, 4, 7, 10, and 13 of the experiment.

PCoA showed that from day 7 onward, community composition was clustered by both nutrient concentration and community size (Fig. 4), showing treatment-driven divergence over time, which was also supported by PERMANOVA results on overall Aitchison distances separately for each time point (Table S1). Differences in community composition were also evident at the genus level on the final day of the experiment (Fig. S4). In small communities, all replicates were consistently dominated by the same three genera, *Rhodoferax, Duganella*, and *Pseudomonas*, which together accounted for 50% or more of the relative abundance. In contrast, large communities exhibited more variation in dominant taxa across replicates. While the low nutrient concentration with large communities had a consistent top-three composition (*Duganella, Limnohabitans*, and *Rhodoferax*), replicates under intermediate and high nutrient concentrations showed divergent dominant genera.

### Aitchison distance and NST

**Figure 5.**
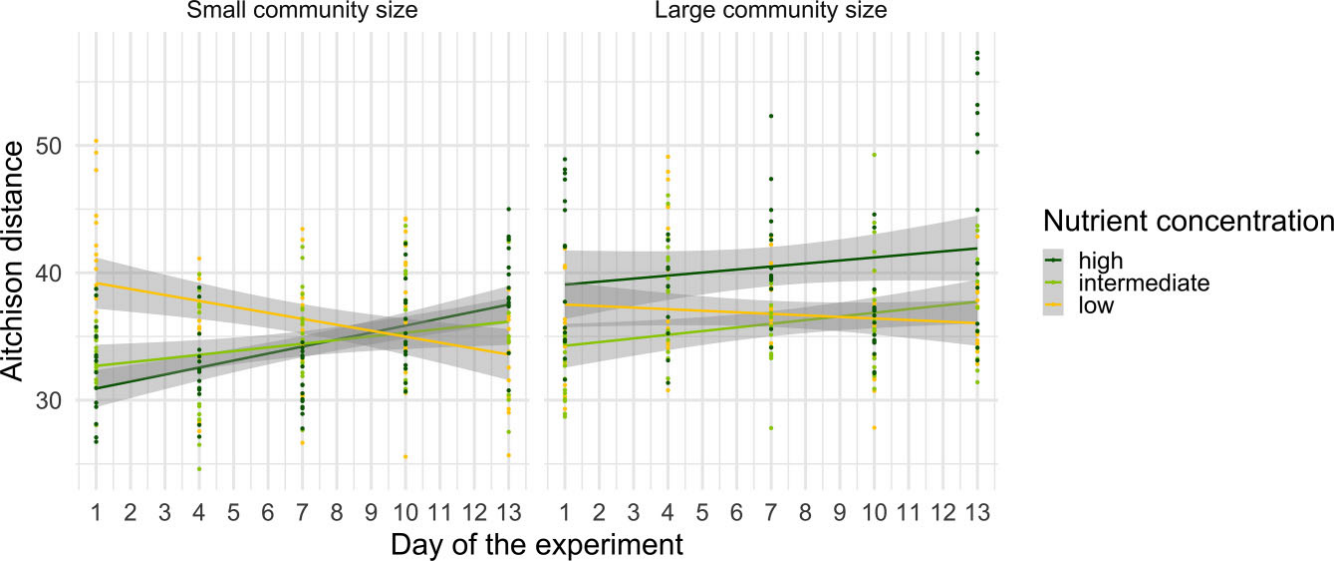
Pairwise Aitchison distances between replicates over time in treatments with different nutrient concentrations (low, intermediate, and high) and community sizes (small, and large). Dots show pairwise Aitchison distances, lines represent linear regression with 95% confidence interval (grey area).

**Figure 6.**
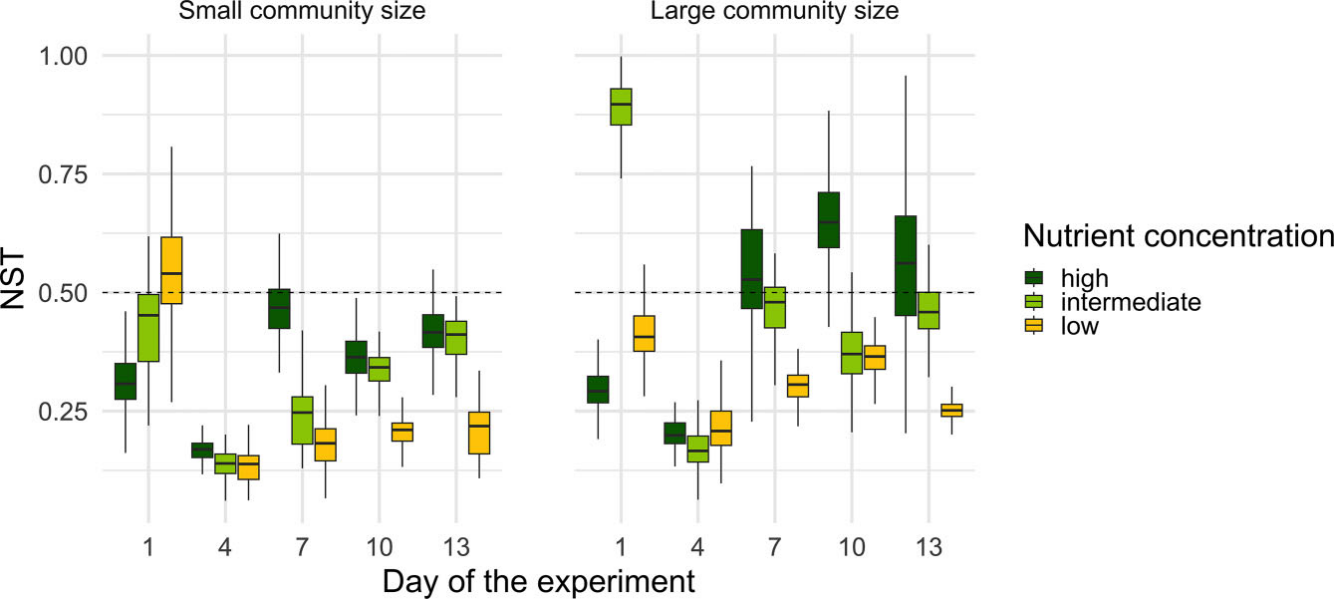
Changes in the NST over time in treatments with different nutrient concentrations (low, intermediate, and high) and community sizes (small, and large). Ranges of NST values for boxplots were retrieved from bootstrap analysis (999 runs). NST estimates the relative importance of stochastic compared to deterministic processes, where values of 0 and 1 represent purely deterministic and stochastic assembled communities, respectively. Hence, values above 0.5 indicate a predominance of stochasticity and values below 0.5 of determinism.

Beta-diversity, measured as pairwise Aitchison distances among replicates within treatments, was significantly affected by nutrient concentration, community size, time, and all interactions among these factors (Table 1). The effect size for the interaction between both treatments and time (χ^2^ = 71) was higher than the effect size of each treatment alone (Table 1). Over time, Aitchison distance increased in intermediate and high nutrient treatments, regardless of community size, whereas it decreased in low nutrient treatments (Fig. 5). By the end of the experiment, the greatest dissimilarity among replicates was observed in large communities under high nutrient concentrations, while the smallest occurred in small communities under low nutrient concentrations (Fig. 5). At all nutrient concentrations, large communities consistently exhibited higher and progressively increasing dissimilarity among replicates over time, whereas small communities showed lower overall dissimilarity that decreased over time. The NST responded significantly to nutrient concentration, community size, time, and their interactions, with the exception of the interaction between nutrient concentration and community size (Table 1). The effect size for the interaction between both treatments and time (χ^2^ = 39) was higher than the effect size of the individual treatments alone (Table 1). NST showed clear temporal patterns, as values were initially high in all treatments but showed an overall decline from day 1 to 4 (Fig. 6). After day 4, NST increased in almost all treatments, specifically in large communities under intermediate and high nutrient concentrations, and reached values above 0.5 in the latter.

**Table 1.** Results of type II Wald chi-square tests from linear mixed-effects models assessing the effects of nutrient concentration, community size, and time point (categorical), and their interactions, on Aitchison distance and NST (Bray–Curtis). The model includes Replicate ID as a random intercept and a first-order autoregressive correlation structure to account for temporal autocorrelation. Significant *P*-values (< .05) are shown in bold.

		Aitchison distance	NST
Nutrient concentration	χ²	20.44	24.56
	Df	2	2
	*P*-value	**< .001**	**< .001**
Community size	χ²	46.88	24.28
	Df	1	1
	*P*-value	**< .001**	**< .001**
Time point	χ²	14.94	98.30
	Df	4	4
	*P*-value	**.005**	**< .001**
Nutrient concentration:community size	χ²	38.66	4.48
	Df	2	2
	*P*-value	**< .001**	0.106
Nutrient concentration:time point	χ²	139.17	84.61
	Df	8	8
	*P*-value	**< .001**	**< .001**
Community size:time point	χ²	68.14	3.74
	Df	4	4
	*P*-value	**< .001**	0.443
Nutrient concentration:community size:time point	χ²	71.17	39.22
	Df	8	8
	*P*-value	**< .001**	**< .001**

## Discussion

We conducted an experimental study to examine the roles of stochastic and deterministic processes in bacterial community assembly across different nutrient concentrations and community sizes. We expected stochasticity to increase over time and to be most pronounced in small communities under high nutrient concentrations, and weakest in large communities at low nutrient concentrations. In agreement with our hypotheses, both higher dissimilarity among replicates and null modelling suggest stochastic processes were more important under intermediate and high compared to low nutrient concentrations, as expected. As richness, abundance, and biomass production were highest in these treatments as well, nutrient availability might have enhanced stochasticity by promoting the growth of rare taxa that might otherwise go extinct (Chase 2010, Huang et al. 2023). In contrast, in low nutrient treatments deterministic processes might have been more important, because stronger selection pressure overruled the effects of stochasticity (Zhou et al. 2010, Stegen et al. 2012). This was also supported by our finding of stronger determinism according to null modelling and lower alpha-diversity (richness and evenness) in the low nutrient treatments indicating determinism, as few species were selected by the resource-poor conditions (Chase and Leibold 2002, Chase 2010). Our results also show that larger communities were more dissimilar among replicates and showed higher levels of stochasticity according to null modelling than smaller ones, which is contrary to our hypothesis as well as results from previous studies, that small communities would be to a greater degree stochastically assembled (Bier et al. 2022a, Siqueira et al. 2020, Pelinson et al. 2022, Jacobi and Siqueira 2023). Moreover, opposite to what we predicted; stochasticity was overall most important in large communities under higher nutrient concentrations. Higher nutrient availability could have weakened resource limitation and reduced competition thus, allowing multiple species to coexist (Chase 2010, Svoboda et al. 2018). This may reflect stronger stochastic variation in initial growth rates, where larger communities, particularly under high nutrient concentrations, allowed a wider range of species to grow rapidly, increasing the chances that different species would dominate early growth in each replicate (Hayashi et al. 2024).

Community composition was shaped by both community size and nutrient concentration, with treatment effects becoming increasingly pronounced over time. These findings are consistent with previous studies showing that both community size (Bier et al. 2022a, Zhou et al. 2014, Ron et al. 2018, Siqueira et al. 2020, Sloan et al. 2021) and nutrient availability (Sadeghi et al. 2021, Jiao et al. 2022, Chen et al. 2023, Huang et al. 2023) can impact microbial community composition. This together with the finding that NST values were in most cases below 0.5 demonstrate the overall importance of deterministic assembly. Importantly, however, our study highlights the potential for complex interactions between community size and nutrient availability over time to influence the role of stochasticity compared to deterministic assembly processes, which few experimental studies have systematically explored.

It was also interesting that the two estimators of bacterial growth that we used in our study gave different results. While bacterial cell abundance increased throughout the entire experiment in large communities under high nutrient concentrations, aligning with findings from previous studies (Chen et al. 2023, Huang et al. 2023), bacterial biomass production peaked at intermediate nutrient concentrations instead. One possible explanation could be that high nutrient concentrations promoted higher cell numbers, but reduced per-cell metabolic activity. Under nutrient-rich conditions, bacterial metabolism may become less efficient, as cells invest proportionally more energy in maintenance respiration or overflow metabolism rather than biomass synthesis (Giorgio and Cole 1998, Sinsabaugh et al. 2016). Moreover, excessive nutrient supply can lead to stoichiometric imbalances; when N or P availability exceeds cellular demands, bacteria may take up and store excess nutrients (‘luxury uptake’) without proportionally increasing carbon incorporation (Sterner and Elser 2002, Geyer and Barrett 2019). Consequently, overall production per cell can decline even as total cell numbers increase. In contrast, intermediate nutrient concentrations may promote optimal microbial growth, as they prevent the physiological stress caused by nutrient limitation at low levels and by inhibitory effects at excessively high levels (Kassen et al. 2000, Geyer and Barrett 2019). Additionally, because cell abundance was measured more frequently than biomass production, it is possible that growth dynamics were captured with greater precision in the abundance data. Future studies should therefore also include measurements of bacterial production at higher frequency to further investigate growth patterns and their relationship with changes in community assembly processes in more detail.

While our study provides important insights into the roles of community size and nutrient availability in shaping microbial assembly, certain methodological constraints may have limited the clarity of the role of stochasticity under these factors. In our study, cell abundances in the small community size treatments could have been too low, thus causing extinction of the majority of species. Implementing a broader gradient of community sizes would therefore allow us to identify the transition zone, where stochasticity and determinism shift, and to test whether this threshold moves under different nutrient availability. The nutrient gradient applied here (5–25 µg/l TP) was narrow relative to severe eutrophication that can be found in some systems, and we therefore encourage future studies to apply broader gradients to better address effects of very high, and also low, nutrient concentrations, respectively. However, our results are valid across a broad range of eutrophication scenarios for many boreal and northern lakes, as the applied TP range captures the transition from oligotrophic to mesotrophic conditions that constitute ecologically meaningful eutrophication scenarios in such lakes (Forsberg and Ryding 1980). Other constraints of our studies relate to that the applied nutrient additions were adjusted following the Redfield ratio and therefore do not take effects of different and more variable elemental ratios (Elser et al. 2007) into account. Moreover, while we used six replicates, which is more compared to most other experimental studies, increasing the number of replicate communities even more could further unravel stochastic and deterministic patterns in the different treatments, for example related to the existence of distinct alternative states of community compositions (Hayashi et al. 2024). Finally, our experiment was run over 13 days and a longer time span should be considered in future experiments to reveal long-term changes in assembly patterns. However, stochasticity is usually most pronounced at the start of the assembly of a community, due to priority effects or ecological drift (Fukami 2015, Dini-Andreote et al. 2015, Bier et al. 2022).

In this study, we applied both pairwise Aitchison distances and null modelling (NST) to evaluate how community size and nutrient concentration influence bacterial community assembly. While Aitchison distance provides a direct measure of compositional dissimilarity between replicate communities (Aitchison 1982), the NST estimates the relative contribution of stochastic versus deterministic processes by comparing observed beta-diversity to randomized null expectations (Ning et al. 2019). However, NST results are known to depend strongly on the choice of null model and its underlying assumptions (Chase et al. 2011, Stegen et al. 2013, Zhou and Ning 2017). Because Aitchison distance-based beta-diversity represents the observed magnitude of within-group compositional variation independently of model assumptions, the discussion of community assembly patterns in this study primarily relies on Aitchison distance results, with NST outcomes used as complementary, model-informed support.

To conclude, our study reinforces previous findings that stochasticity tends to become more important under high nutrient availability, likely due to enhanced bacterial growth and reduced competitive exclusion. Importantly, we also demonstrate that larger communities are more strongly influenced by stochastic processes than smaller ones, suggesting that stochastic initial growth of species may play a greater role when community size is larger. Unlike many previous studies that focus on single environmental drivers, our experimental design allowed us to investigate the effects of community size and nutrient availability and their interactions on assembly processes. These findings contribute novel insights into how multiple ecological factors interactively shape bacterial communities. Managing both nutrient inputs and secondary stressors that reduce microbial biomass may therefore be critical to maintaining diverse and functionally resilient microbial communities. Future work should build on this approach to further disentangle the complex relationships between environmental change and stochastic community assembly. Expanding this research will help to refine ecological theory and enhance our ability to manage microbial communities under environmental stress.

## Supplementary Material

fiaf110_Supplemental_File

## Data Availability

Sequencing data have been deposited at the European Nucleotide Archive (ENA) under the project number PRJEB96060. Bacterial abundance and production data are available on Zenodo: 10.5281/zenodo.16911903.
