# Supplementary material for: Linking nutrient availability and community size to stochasticity in microbial community assembly
Source: FEMS Microbiol Ecol. 2025 Oct 28;101(12):fiaf110. doi: 10.1093/femsec/fiaf110 (PMC12603558; doi:10.1093/femsec/fiaf110)
Supplement: fiaf110_Supplemental_File [file fiaf110_supplemental_file.docx]

Supplementary Information

**Linking productivity and community size to stochasticity in microbial community assembly**

Berenike Bick, Theresa Lumpi, Eva S Lindström, Silke Langenheder

Supplementary Methods

Supplementary Methods S1: Library preparation

Library preparation was carried out in a two-step PCR protocol. DNA was first amplified in triplicates per sample (with a volume of each 20 µl), with 1µl template DNA and 19 µl reaction mixture: 4 µl of 5xQ 5 reaction buffer (New England Biolabs, Ipswich, MA, USA), 0.5 µl of 10 µM custom Illumina adapter-N4-341 F (IDT, Coralville, Iowa, USA) and 0.5 µl of 10 µM reverse primer custom Illumina adapter-805NR (IDT, Coralville, Iowa, USA), 2 µl of 2 mM dNTPs (Invitrogen, Waltham, MA, USA), 0.2 µl of 2 U/µl of Q5 HF DNA polymerase (New England Biolabs, Ipswich, MA, USA) and 11.8 µl of nuclease-free DEPC treated water (Invitrogen, Waltham, MA, USA). The reaction was run on a thermocycler (LifePro, BIOER, China) for 3 min at 98°C for initial denaturation, followed by 20 cycles of each 10 s at 98°C for dissociation, 30 s at 48°C for annealing, 20 s at 72°C 20 for elongation and 2 min at 72°C for a final extension. The amplicons were subsequently analysed by agarose gel electrophoresis to verify the presence of PCR products and thereafter stored at 6°C or -20°C for future use. For purification, triplicates were pooled and magnetic beads (magtivio, HK Nuth, Netherlands) used to remove unwanted components from the reaction mixture. Before the second PCR-step, DNA concentrations were measured and diluted with nuclease-free DEPC treated water to similar DNA concentrations between samples.

In the second PCR sample-specific barcodes were attached to each end of the amplicon. For that, reactions were run in singlets (with a total volume of 20 µl), with 2 µl of template from the 1^st^ PCR and 18 µl of reaction mixture: 4 µl of 5xQ 5 reaction buffer, 1µl of 5 µM custom forward index i5, illu-N501- N508 (IDT, Coralville, Iowa, USA) and 1 µl of 5 µM custom reverse index i7, illu-N701- N712 (IDT, Coralville, Iowa, USA), 2 µl of 2 mM dNTPs and 9.8 µl of nuclease-free DEPC-treated water. Both PCR steps were run on the same thermocycler with a similar program, but in the second PCR, reactions were reduced to 15 cycles, initial denaturation time reduced to 30 s and annealing temperature was increased to 66 °C.

After the second PCR step, PCR products were analysed with Agarose gel electrophoresis and purified with magnetic beads. In order to pool all samples in equal molecular amounts, DNA concentration was measured with a PicoGreen assay. The pooled amplicons were thereafter purified again with gel purification (Promega, Madison, WI, USA) before delivering to the sequencing facility.

Supplementary Methods S2: Bacterial carbon production

Bacterial carbon production was measured by leucine (^3^H) incorporation into bacteria protein. For that, samples were taken at five time points throughout the experiment (day 1, 4, 7, 10 and 13 of the experiment). Samples and blanks were then incubated with a leucine mix of L-[3, 4, 5 -^3^H] (Perkin Elmer) for one 1 hour in the dark, after which the incubation was stopped by adding 100% Trichloroacetic acid (TCA) and samples stored at 4°C. The following day, samples were washed in several steps with 5% TCA and 96% Ethanol, a scintillation cocktail was added and samples were stored at room temperature. Disintegrations per minute of all samples were measured the following day with a liquid scintillation counter (Hidex 600 SL, counting time: 300 sec., coincidence time: 35 ns) and recalculated into carbon production rates.

Supplementary Tables

Table S1: Type II Wald χ² results from linear mixed-effects models testing the effects of nutrient concentration, community size, and their interaction on bacterial cell abundance, carbon production ASV richness and Pielou evenness. The model included a random intercept for Replicate ID and a first-order autoregressive (AR1) correlation structure for residuals across experimental days within replicates. Reported are the Wald χ² statistic (χ²), numerator degrees of freedom (df), and P-values.

|  | **Nutrient concentration** | | | **Community size** | | | **Nutrient concentration:Community size** | | |
| --- | --- | --- | --- | --- | --- | --- | --- | --- | --- |
|  | χ² | Df | p-value | χ² | Df | p-value | χ² | Df | p-value |
| Cell abundance | 5.97 | 2 | 0.051 | 2.80 | 1 | 0.094 | 0.02 | 2 | 0.992 |
| Carbon production | 59.60 | 2 | **< 0.001** | 27.79 | 1 | **< 0.001** | 1.05 | 2 | 0.591 |
| ASV richness | 9.56 | 2 | **0.008** | 9.85 | 1 | **0.002** | 4.42 | 2 | 0.110 |
| Pielou evenness | 142.02 | 2 | **< 0.001** | 4.97 | 1 | **0.026** | 25.94 | 2 | **< 0.001** |

Table S2: Permutational multivariate analysis of variances (PERMANOVA) results for principal coordinate analysis (PCoA) for each day of the experiment separately (day 1, 4, 7, 10 and 13) testing effects of nutrient concentration and community size on overall Aitchison distances.

|  | **Nutrient concentration** | | | **Community size** | | |
| --- | --- | --- | --- | --- | --- | --- |
|  | F | Df | p-value | F | Df | p-value |
| Day 1 | 2.72 | 2 | **< 0.001** | 7.28 | 1 | **< 0.001** |
| Day 4 | 4.54 | 2 | **< 0.001** | 7.88 | 1 | **< 0.001** |
| Day 7 | 8.61 | 2 | **< 0.001** | 9.28 | 1 | **< 0.001** |
| Day 10 | 8.43 | 2 | **< 0.001** | 9.13 | 1 | **< 0.001** |
| Day 13 | 8.65 | 2 | **< 0.001** | 9.09 | 1 | **< 0.001** |

Supplementary Figures


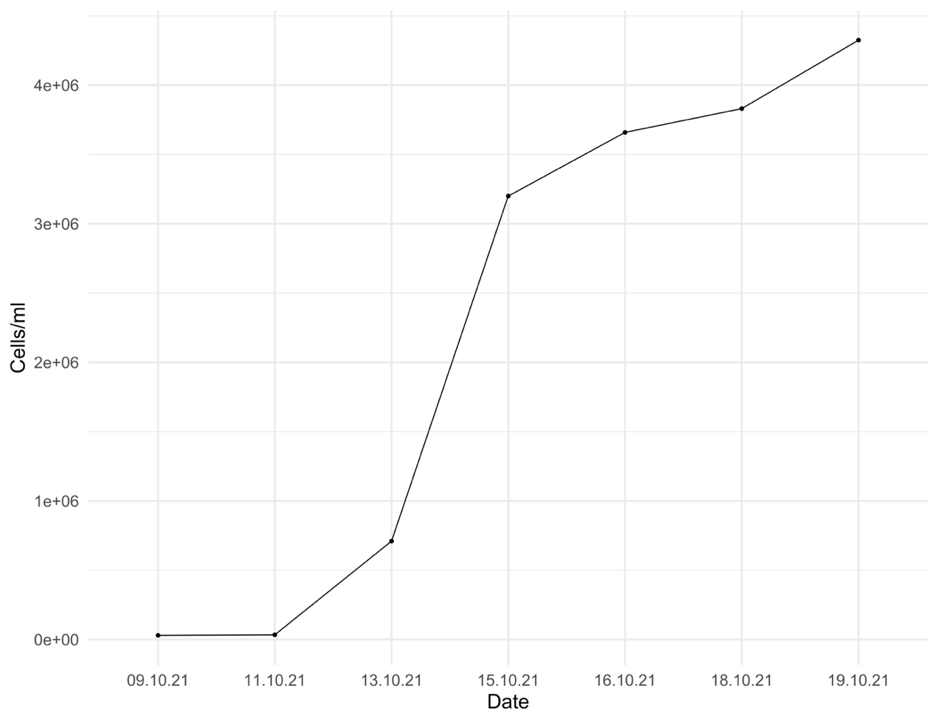


Figure S1: Growth of inoculum prior to start of the experiment over time in cells/ml.


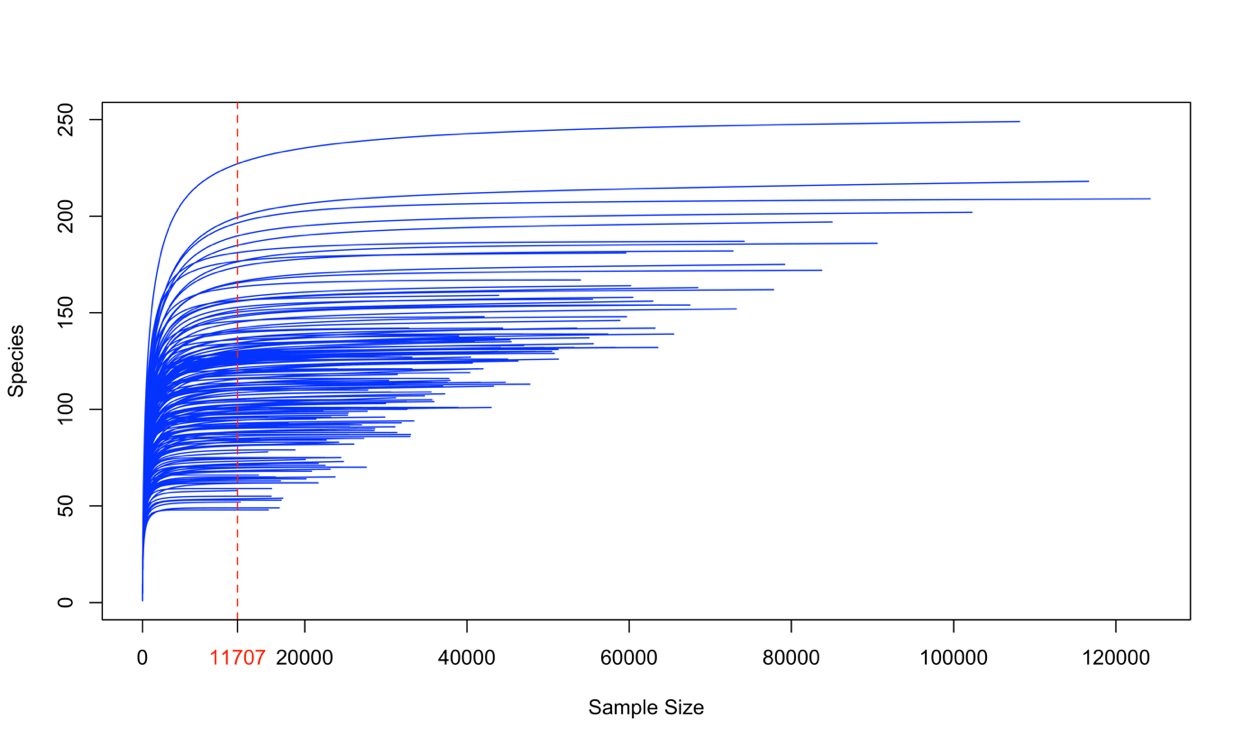


Figure S2: Rarefaction curves of all samples (n=178), the red line shows the read number to which the samples were rarefied.


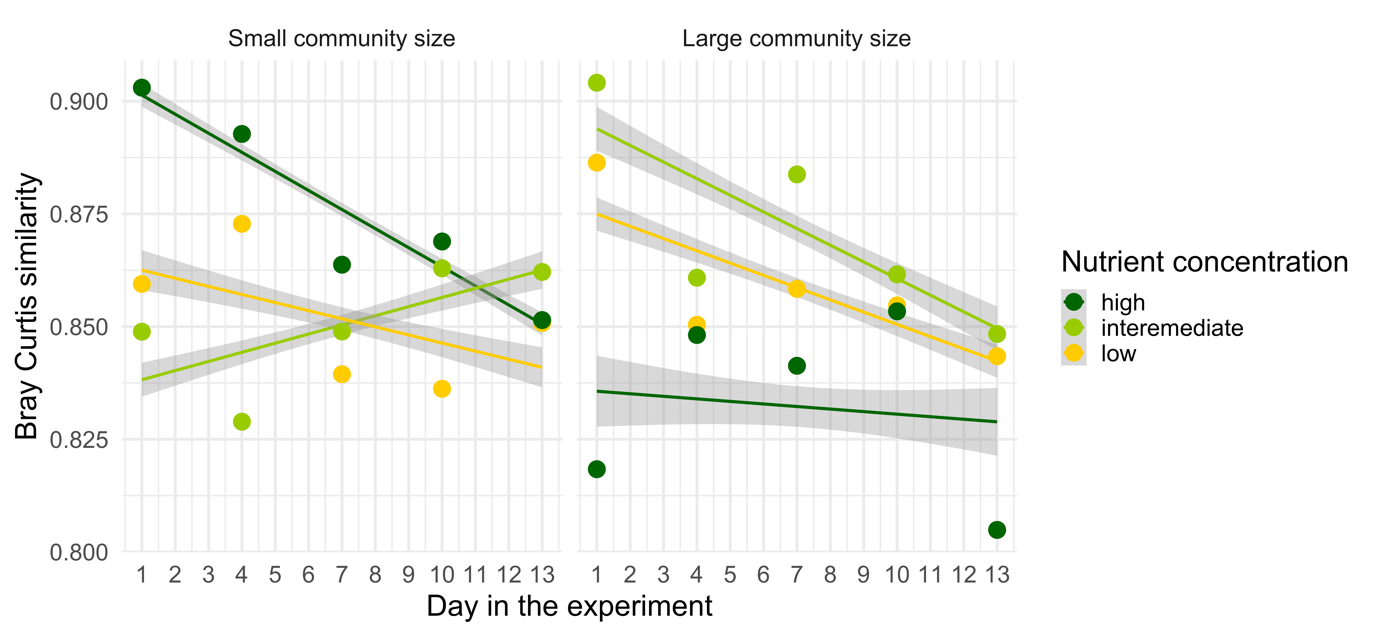


Figure S3: Bray Curtis similarity over time for each time point represented by a dot and a fitted line for small (left) and large community size (right), colours indicate nutrient concentrations (dark green represents high, light green intermediate and yellow low nutrient concentration).


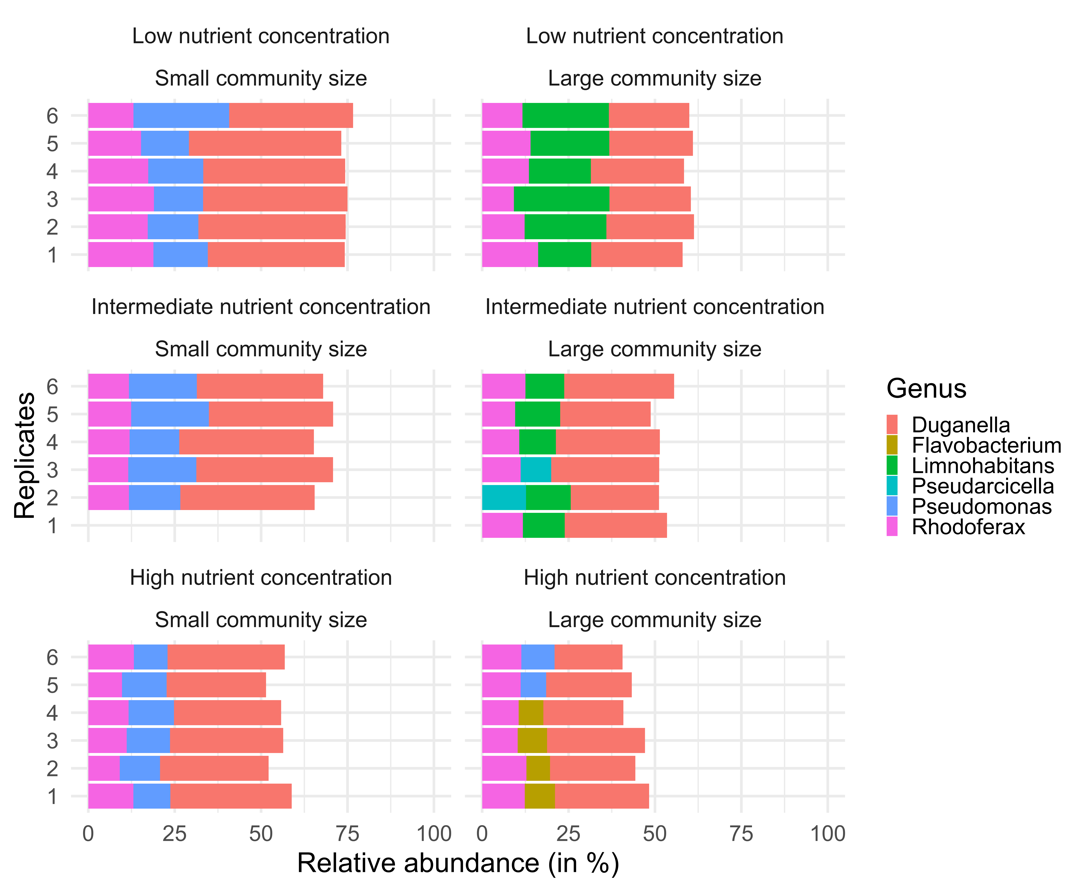


Figure S4: Relative abundance (in %) of top 5 taxa in each replicate microcosm represented with bars, top plots show low, middle plots intermediate and bottom plots high nutrient concentration, small community size treatments are presented on the left and large on the right side, colours indicate different genera.


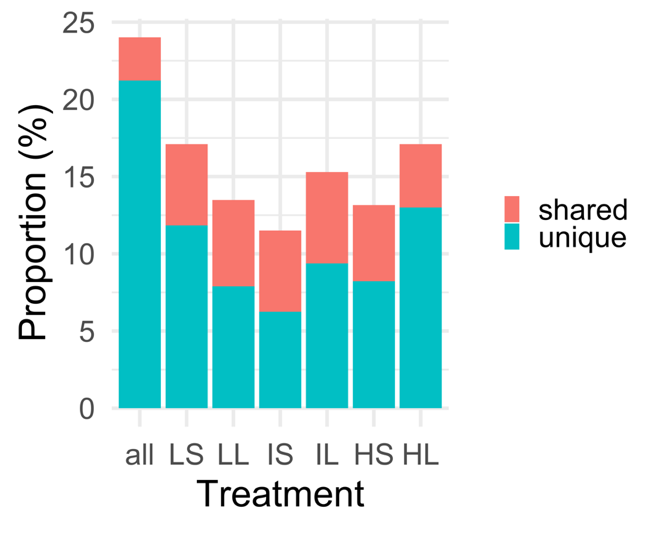


Figure S5: Proportion (in %) shown in bars of shared (red) and unique (green) taxa of all data points, low nutrient concentration with small (LS) and large community size (LL), intermediate nutrient concentration with small (IS) and large community size (IL) and high nutrient concentration with small (HS) and large community size (HL).
